# A study of minimum segment width parameter on VMAT plan quality, delivery accuracy, and efficiency for cervical cancer using Monaco TPS

**DOI:** 10.1002/acm2.12422

**Published:** 2018-07-30

**Authors:** Yuanyuan Wang, Li Chen, Fengying Zhu, Wanjing Guo, Dandan Zhang, Wenzhao Sun

**Affiliations:** ^1^ Department of Radiation Oncology Hefei Ion Medical Center Hefei China; ^2^ State Key Laboratory of Oncology in South China Collaborative Innovation Center for Cancer Medicine Sun Yat‐sen University Cancer Center Guangzhou China; ^3^ Shunde Hospital of Southern Medical University Shunde China; ^4^ Qingyuan People's Hospital Qingyuan China

**Keywords:** cervical cancer, delivery time, Minimum segment width, plan quality, VMAT

## Abstract

**Purpose:**

The purpose of this study was to study the influence of the minimum segment width (MSW) on volumetric modulated arc therapy (VMAT) plan quality, delivery accuracy, and efficiency for cervical cancer treatment.

**Methods:**

Nineteen patients with cervical cancer were randomly selected to design VMAT plans. Three VMAT plans were generated for each patient incorporating MSWs of 0.5, 1.0, and 1.5 cm while other planning parameters remained constant using the Monaco treatment planning system (TPS) with 6 MV X rays delivered from an Elekta Synergy linear accelerator. Plan quality and delivery efficiency were evaluated based on dose‐volume histograms (DVHs), control points, monitor units (MUs), dosimetric measurement verification results, and plan delivery time.

**Results:**

Except for the small difference in target dose coverage and maximum dose, there were no statistically significant differences between the other dosimetric parameters in the planning target volumes. The 1.0 and 1.5 cm MSW plans showed lower maximum doses to the spinal cord than the 0.5 cm plan; doses to other organs at risks were similar regardless of MSWs. The mean reductions of total MUs when compared with the 0.5 cm plan were 14.5 ± 6.1% and 20.9 ± 7.9% for MSWs of 1.0 and 1.5 cm, respectively. The calculated gamma indices using the 3% and 3 mm criteria were 96.2 ± 0.6%, 97.0 ± 0.6%, and 97.6 ± 0.6% for the 0.5, 1.0 and 1.5 cm MSW plans, respectively. The plan delivery times decreased with increasing MSWs (*p *<* *0.05).

**Conclusion:**

Increasing the MSW allows for improved plan delivery accuracy and efficiency without significantly affecting the VMAT plan quality. MSWs of 1.0 and 1.5 cm improved the plan quality, delivery accuracy, and efficiency for cervical VMAT radiation therapy.

## 1 INTRODUCTION

Over the last decade, volumetric modulated arc therapy (VMAT) has been explored and implemented to treat a variety of cancers including in the prostate, head and neck, lung, and spine.^1^, ^2^, ^3^, ^4^ VMAT is a dynamic treatment technique in which the radiation dose rates, gantry speeds, and movements of the multi‐leaf collimator and jaws are simultaneously varied while the beam is on.^5^ VMAT enables greater dose conformity to target tissues, and spares more of the normal tissue than traditional three‐dimensional conformal radiation therapy (3D‐CRT) and intensity‐modulated radiation therapy (IMRT).^6^, ^7^, ^8^ Generally, VMAT planning involves a two‐step optimization procedure: First, ideal fluence maps are optimized and calculated according to an optimization algorithm; next, the arc sequencer algorithm converts these fluence maps to arc delivery maps while optimizing the multi‐leaf collimator shape sequence to serial segments (control points). The minimum segment width (MSW) parameter takes an important role in the creation of the shapes and sizes of these segments. When designing VMAT plans to treat cervical cancer, optimization often results in some long and narrow segments that may have a notable impact on plan delivery, and can sometimes lead to a low verification passing rate and even an interruption. The impact of VMAT planning parameters, such as small monitor unit (MU) per segment, dose rate, and control point spacing, on plan quality have been evaluated for a Pinnacle^3^ treatment planning system (TPS) using the Elekta Synergy/Varian Trilogy linear accelerator.^9^, ^10^ However, there have been no reports regarding MSW optimization in terms of VMAT plan quality, delivery, accuracy, and efficiency. The purpose of this study was to explore the influence of the MSW parameter on the quality and delivery accuracy of VMAT plans for cervical cancer to provide a useful reference for clinical treatment planning.

## 2 MATERIALS AND METHODS

### 2.A Patient selection

Nineteen patients with cervical cancer aged between 38 and 78 yr (average 52.6 yr) who underwent VMAT at our hospital between June 2017 and October 2017 were enrolled in this study. This study was approved by the Ethical Commission of our cancer center. Because this was not a treatment‐based study, our institutional review board waived the need for written informed consent from the participants. The patient information was anonymized and de‐identified to protect patient confidentiality.

### 2.B Simulation and contouring

**Figure 1:**
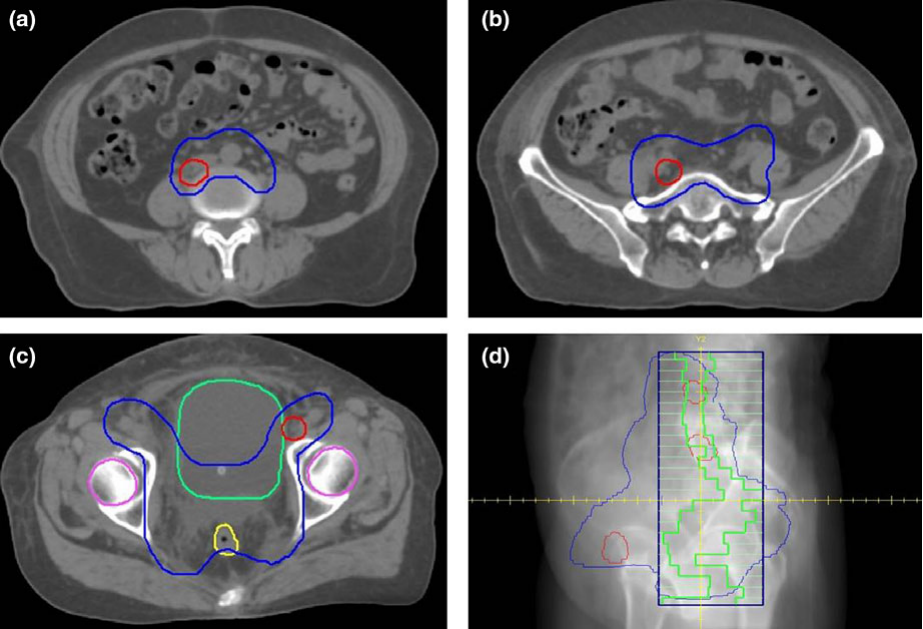
The shapes of the planning target volumes (a–c) and a typical segment (d) for a cervical volumetric modulated arc therapy plan. The red contour denotes the PTV60 and the blue contour denotes the PTV45 in (a), (b), and (c). d: Digitally reconstructed radiography for a typical segment from the beam's eye view (gantry = 112°). The green outline represents the shape of the segment.

All patients were immobilized with a vacuum bag system with a supine position, and were then scanned using a Philips computed tomography (CT) simulator with a slice thickness of 3 mm. The reconstructed CT images were transmitted to Monaco 5.11 TPS. Gross tumor volume (GTV) and clinical tumor volume (CTV) were delineated on CT images by an experienced radiation oncologist according to the institutional protocol. A contour expansion was applied to the GTV and CTV to delineate a planning target volume (PTV) that would receive 60 Gy (PTV60) and 45 Gy (PTV45). PTV60 was derived from the GTV with involved lymph nodes plus a uniform 5 mm margin, while the PTV45 was generated from the CTV plus a uniform 6–8 mm margin (Fig. 1). The bladder, rectum, spinal cord, kidneys, and femoral heads were delineated as organs at risk (OARs).

### 2.C Treatment planning

For all patients, VMAT plans were designed using the Monaco TPS via the Monte Carlo (MC) algorithm, and plans were delivered using the Elekta Synergy linear accelerator with X ray beam energy (6 MV). Each case was planned with a single arc of 360° rotating clockwise from 181° to 179°. The collimator angle for each patient was fixed to 0° during gantry rotation, based on the patient's anatomy. The statistical uncertainty of the MC algorithm was 3% per control point, and the final dose was calculated with a calculation grid resolution of 3 mm. The maximum number of control points was 150 for each plan. Three VMAT plans, 0.5 cm MSW, 1.0 cm MSW, and 1.5 cm MSW, were generated with MSWs of 0.5, 1.0, and 1.5 cm, respectively, while other parameters and cost functions remained unchanged. The prescription dose was the dose to 98% of the PTV60 (D98%) that received at least 60 Gy in 23 fractions. The cost functions are displayed in Table 1.

**Table 1:** The cost functions of VMAT planning for cervical cancer

ROIs	Cost function	Parameter	Iso constraint
PTV60	Target penalty	95%	60 Gy
	Underdose DVH	60 Gy	98%
	Quadratic overdose	63 Gy	20
PTV45	Target penalty	95%	45 Gy
	Underdose DVH	45 Gy	96%
	Quadratic Overdose	48 Gy	50
Spinal cord	Maximum dose	NA	40 Gy
Rectum	Quadratic overdose	45 Gy	40
Bladder	Quadratic overdose	45 Gy	60
Kidney‐L	Serial	k = 12	18 Gy
Kidney‐R	Serial	k = 12	18 Gy
Femoral head‐L	Maximum dose	NA	48 Gy
Femoral head‐R	Maximum dose	NA	48 Gy
Body	Quadratic overdose	45 Gy	20
	Quadratic overdose	30 Gy	120
	Maximum dose	Shrink 0.9 cm	45 Gy

ROI, region of interest; PTV, planning target volume; DVH, dose‐volume histogram.

### 2.D Plan evaluation

The different MSW cervical plans were compared in terms of dosimetric indices such as the homogeneity index (HI), conformity index (CI), maximum dose of target volume, target coverage (TC), MUs, control points, and the DVH parameters concerning OARs. The TC and HI were determined as follows:

(1) $$\text{TC}\left(\%\right)=\left({\text{TV}}_{\text{PI}}/\text{TV}\right)\times 100$$

(2) $$\text{HI}={\text{D}}_{5\%}/{\text{D}}_{95\%}$$

where TV_PI_ represents the target volume receiving the prescription dose, TV represents the total target volume. D_5%_ is the minimum dose received by 5% of the PTV according to the DVH (indicating the maximum dose), and D_95%_ is the minimum dose received by 95% of the PTV (indicating the minimum dose). A lower HI represents better homogeneity.

The CI was calculated as below:

(3) $$\text{CI}={\left({\text{TV}}_{\text{PI}}\right)}^{2}/\left(\text{TV}\times {\text{V}}_{\text{PI}}\right)$$

where V_PI_ represents the total volume receiving the prescription dose (60 Gy or 45 Gy); the closer the CI is to 1, the more conformal is the target dose distribution.

Absolute dose distributions were measured using an Elekta iViewGT aSi electronic portal imaging device (EPID) detector. The EPID has a sensitive area that is 41 cm × 41 cm in size with an effective pixel size of 0.04 cm × 0.04 cm. Offset, gain, and pixel corrections were performed for each image, and a time‐integrated signal was obtained for every plan. The pixel values in the EPID images were reconstructed to dose values at a source to axis distance of 100 cm in the phantom.^11^ The measured and computed doses were analyzed using RapiDose (Version 2.1, RayDose Inc., China) commercial software to analyze and calculate the gamma passing rate (GPR).^12^ The plan delivery time (PDT; ie, the interval between beam activation and deactivation) was measured simultaneously for each plan.

### 2.E Statistical analysis

The paired *t*‐test followed by Bonferroni's correction was applied in the intergroup comparison for dosimetric parameters and measurement results using the SPSS 19.0 software. A *p‐*value <0.05 indicated a statistically significant difference.

## 3 RESULTS

### 3.A Target doses

**Figure 2:**
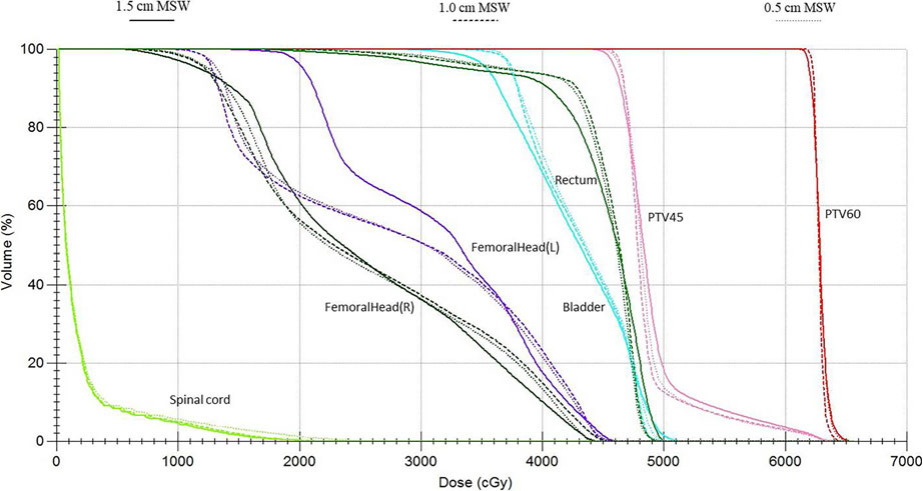
The dose‐volume histograms of three volumetric modulated arc therapy plans with different minimum segment widths (MSWs) for a typical cervical cancer.

The target doses of the three VMAT plan groups are shown in Table 2. The mean and maximum PTV60 and PTV45 doses were not markedly different among the three plans. The target dose coverage of the plan using an MSW of 0.5 cm was higher than that of the plan using an MSW of 1.0 cm, which in turn was better than that of the plan using an MSW of 1.5 cm. The DVH results using these three plans in a typical patient with cervical cancer are shown in Fig. 2. The maximum PTV60 and PTV45 doses of the plan using an MSW of 1.5 cm were higher than those of the plans with MSWs of 0.5 and 1.0 cm.

**Table 2:** PTV dosimetric results of the VMAT plans used to treat 19 cervical cancer patients devised using three different MSWs

PTV	Parameter	0.5 cm MSW	1.0 cm MSW	1.5 cm MSW	*p*_1_	*p*_2_	*p*_3_
PTV60	TC (%)	99.5 ± 0.13	99.4 ± 0.16	99.18 ± 0.22	0.02	0.01^a^	0.04
	D_mean_ (Gy)	62.7 ± 0.06	62.6 ± 0.05	62.73 ± 0.05	0.07	0.38	0.08
	D_max_ (Gy)	65.4 ± 0.23	65.2 ± 0.19	65.52 ± 0.26	0.01^a^	0.68	0.07
	CI	0.44 ± 0.02	0.51 ± 0.06	0.47 ± 0.02	0.27	0.98	0.27
	HI	1.04 ± 0.00	1.04 ± 0.00	1.04 ± 0.00	0.33	0.86	0.87
PTV45	TC (%)	99.45 ± 0.12	99.28 ± 0.13	98.88 ± 0.16	0.02	0.01^a^	0.01^a^
	D_mean_ (Gy)	49.76 ± 0.39	49.72 ± 0.39	49.82 ± 0.41	0.26	0.16	0.02
	D_max_ (Gy)	65.66 ± 0.28	65.37 ± 0.23	65.51 ± 0.22	0.01^a^	0.782	0.11
	CI	0.75 ± 0.01	0.75 ± 0.01	0.75 ± 0.01	0.37	0.54	0.92
	HI	1.27 ± 0.02	1.26 ± 0.02	1.28 ± 0.02	0.02	0.01^a^	0.00^a^

*p*_1_, *p*‐value comparing 0.5 cm and 1.0 cm MSW plans; *p*_2_, *p*‐value comparing 0.5 and 1.5 cm MSW plans; *p*_3_, *p*‐value comparing 1.0 and 1.5 cm MSW plans. PTV, planning target volume; VMAT, volumetric modulated arc therapy; MSW, minimum segment width; TC, target coverage; D_mean_, mean dose; D_max_, maximum dose; CI, conformity index; HI, heterogeneity index.

a statistically significant according to Bonferroni correction.

**Figure 3:**
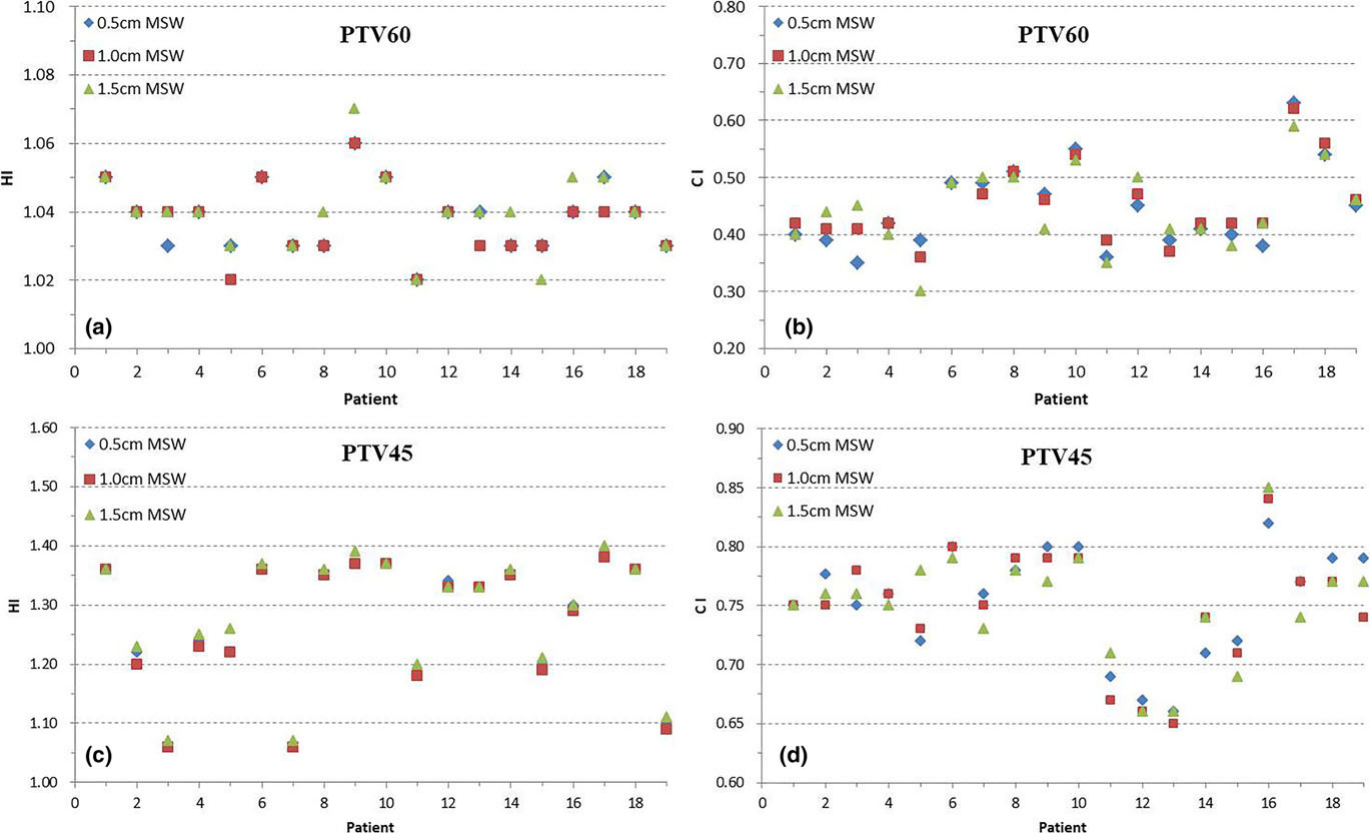
The homogeneity index (HI) and conformity index (CI) of the planning target volumes (PTVs) for 19 cervical volumetric modulated arc therapy plans using three different minimum segment widths (MSWs). a: HI of PTV60; b: CI of PTV60; c: HI of PTV45; d: CI of PTV45.

The CI and HI values for all treatment plans are shown in Fig. 3. The CI and HI for the PTV60 are similar among all three group plans (*p *>* *0.05). As for the PTV45, the 1.0 cm MSW plan had a lower HI than the 0.5 and 1.5 cm MSW plans.

### 3.B OAR dose

OAR dose results are shown in Table 3. Except for the lower maximum dose to the spinal cord when using the plan with the highest MSW, there were no significant differences between the three types of VMAT plans in terms of doses to the remaining OARs.

**Table 3:** Doses to the OARs of the VMAT plans with three different MSWs for 19 cervical cancer patients

OAR	Parameter	0.5 cm MSW	1.0 cm MSW	1.5 cm MSW	*p*_1_	*p*_2_	*p*_3_
Spinal cord	D_max_ (Gy)	25.18 ± 3.89	24.28 ± 3.79	23.13 ± 3.59	0.08	0.01^a^	0.02
Rectum	V_30 Gy_ (%)	8.96 ± 2.25	8.43 ± 2.19	9.01 ± 2.20	0.15	0.55	0.06
Bladder	V_30 Gy_ (%)	12.49 ± 3.16	11.73 ± 2.79	12.21 ± 2.97	0.12	0.83	0.15
Kidney‐L	V_45 Gy_ (%)	62.10 ± 5.40	63.53 ± 5.44	61.51 ± 5.58	0.28	0.92	0.16
Kidney‐R	V_45 Gy_ (%)	48.38 ± 4.10	47.62 ± 4.04	48.52 ± 4.24	0.42	0.74	0.66
Femoral head‐L	V_30 Gy_ (%)	38.91 ± 2.63	39.32 ± 2.75	41.68 ± 3.56	0.75	0.14	0.07
Femoral head‐R	V_30 Gy_ (%)	37.56 ± 3.73	40.77 ± 3.91	39.87 ± 3.98	0.06	0.36	0.67

*p*_*1*_, *p*‐value comparing 0.5 and 1.0 cm MSW plans; *p*_2_, *p*‐value comparing 0.5 and 1.5 cm MSW plans; *p*_*3*_, *p*‐value comparing 1.0 and 1.5 cm MSW plans. VMAT, volumetric modulated arc therapy; OAR, organ‐at‐risk; MSW, minimum segment width; D_max_, maximum dose; V_x_, percentage volume of region of interest receiving at least X Gy.

a statistically significant according to Bonferroni correction.

### 3.C Control points and MUs

**Figure 4:**
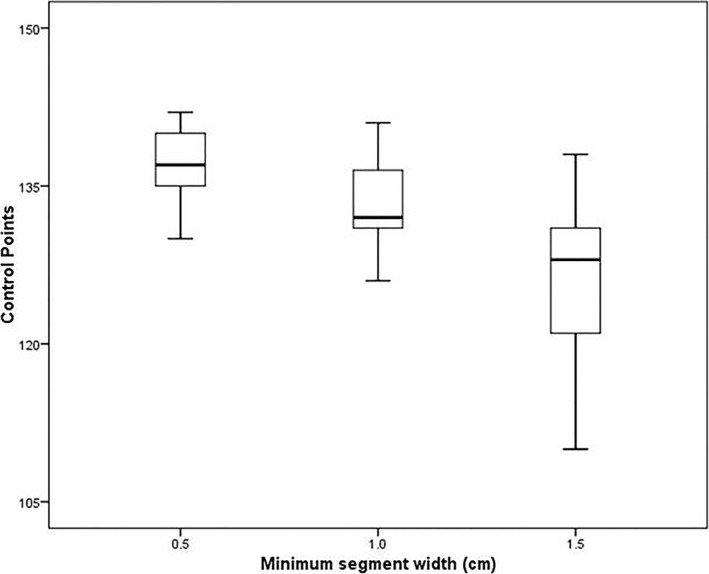
Box and whisker histograms of the control points for the different minimum segment widths plans.

**Figure 5:**
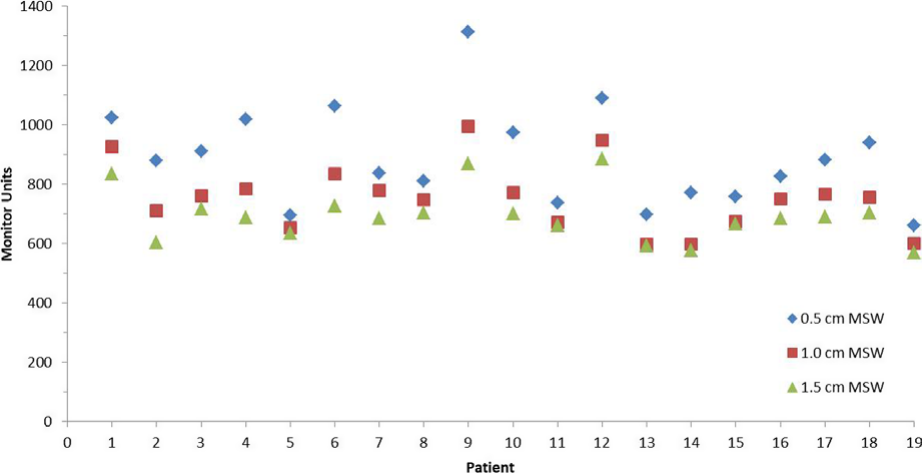
Monitor units for plans with different minimum segment width (MSW).

As the MSW value increased, the control points of the cervical cancer VMAT plan decreased; the mean number of control points for the plans with MSWs of 0.5, 1.0 and 1.5 cm were 137, 133, and 125, respectively (Fig. 4). Moreover, the MUs of the VMAT plan decreased as the MSW increased (Fig. 5); the mean MUs for the plans with MSWs of 0.5, 1.0, and 1.5 cm were 889.1 ± 164.5, 754.3 ± 113.4, and 694.1 ± 88.8, respectively.

### 3.D Dosimetric verification and plan delivery time

Comparison between the measured planar dose and TPS‐calculated dose was analyzed using the gamma passing criteria of a 2% dose difference (DD) and a 2 mm distance to agreement (DTA), as well as with passing criteria of a 3% DD and 3 mm DTA. Table 4 shows the GPRs for the 0.5, 1.0, and 1.5 cm MSW plans. The GPR was highest with the plan using a MSW of 1.5 cm and lowest in the plan using a MSW of 0.5 cm. Table 4 also shows the plan delivery time from beam turn‐on to turn‐off for all 19 patients. As the MSW increased, the control points and MUs of the VMAT plan decreased, as did the plan delivery time.

**Table 4:** Gamma passing rates and delivery times for plans with different minimum segment widths (MSWs)

Parameter	0.5 cm MSW	1.0 cm MSW	1.5 cm MSW	*p*_1_	*p*_2_	*p*_3_
3% and 3 mm GPR	96.23 ± 0.59	97.00 ± 0.56	97.59 ± 0.59	<0.01^a^	<0.01^a^	<0.01^a^
2% and 2 mm GPR	85.35 ± 1.38	87.58 ± 1.27	89.28 ± 1.44	<0.01^a^	<0.01^a^	0.01^a^
PDT (min)	4.39 ± 0.12	4.23 ± 0.10	4.07 ± 0.11	0.01^a^	<0.01^a^	0.01^a^

*p*_*1*_, *p*‐value comparing 0.5 cm and 1.0 cm MSW plans; *p*_2_, *p*‐value comparing 0.5 cm and 1.5 cm MSW plans; *p*_*3*_: *p*‐value comparing 1.0 cm and 1.5 cm MSW plans. MSW, minimum segment width; GPR, gamma passing rate; PDT, plan delivery time.

a statistically significant according to Bonferroni correction.

## 4 DISCUSSION

Designing VMAT plans for treating cervical cancer produces a large number of long, small, and irregular segments. MSW takes an important role in the forming of optimized apertures. These segments sometimes lead to low verification rate and even interruption during delivery of VMAT plan in clinical works. As plan complexity can be reduced by increasing the MSW, we compared the qualities of three different cervical cancer VMAT plan optimization schemes that were based on three different MSW values. Quality comparisons included evaluating the HI, CI, TC, maximum doses, and mean doses to the PTV, as well as the dose‐volume index of the OARs, MUs, and control points. VMAT plans generated with a MSW of 1.0 cm were found to have similar dose distributions as plans with MSWs of 0.5 cm. However, plans with MSWs of 1.5 cm were of slightly worse quality, although they still satisfied clinical requirements (Fig. 2).

The measured and computed doses were analyzed using an EPID detector. All treatment plans showed good GPRs; the mean GPR was >94% when using the 3% DD and 3 mm DTA criteria, and >85% when using the 2% DD and 2 mm DTA criteria.^13^ This showed that the measured dose was consistent with the calculated dose. The dose measured when using a higher MSW showed better agreement with the calculated dose from the TPS; this was expected given that the number of small fields decrease as the MSW increased, and dosimetric verification would therefore be relatively easier.

In addition, the number of control points and MUs decreased as the MSWs increased. When compared to the plan using a MSW of 0.5 cm, the mean MU reductions in the plans using MSWs of 1.0 and 1.5 cm were 15.2% and 21.9%, respectively, while the total control points were decreased by 2.9% and 8.8%, respectively. Previous studies showed that decreasing the MUs for treatment delivery reduces the constraint factor of the leaves’ trajectories, complexity of intensity‐modulated radiation therapy plans, and treatment time.^14^, ^15^, ^16^, ^17^ Hence, as the MSW increases and VMAT plan complexity decreases, the therapeutic efficiency may improve as well. The average delivery times of the plans using MSWs of 1.0 and 1.5 cm were decreased by 9.6 and 19.2 s, respectively (a drop of approximately 3.6% and 7.3%, respectively), compared to the plan with a MSW of 0.5 cm.

## 5 CONCLUSION

Generally, VMAT plans of cervical cancer that are generated with smaller MSWs have not only increased target coverage and conformal index, but also lead to more control points and MUs that would produce lower GPRs and greater treatment delivery times. Our data indicated that VMAT plans with MSWs of 1.0 cm show a clear advantage in terms of a trade‐off between plan quality and delivery efficiency for cervical cancer, and can optimally meet the clinical requirements.

## CONFLICTS OF INTEREST

The authors declare no conflicts of interest.
